# The effects of epidermal fatty acid profiles, 1-oleoglycerol, and triacylglycerols on the susceptibility of hibernating bats to *Pseudogymnoascus destructans*

**DOI:** 10.1371/journal.pone.0187195

**Published:** 2017-10-27

**Authors:** Melissa R. Ingala, Rebecca E. Ravenelle, Johanna J. Monro, Craig L. Frank

**Affiliations:** 1 Department of Biological Sciences, Fordham University, Louis Calder Center, Armonk, NY, United States of America; 2 Environmental Science Program, Fordham University, Bronx, NY, United States of America; CSIRO, AUSTRALIA

## Abstract

White Nose Syndrome (WNS) greatly increases the over-winter mortality of little brown (*Myotis lucifugus*), Indiana (*M*. *sodalis*), northern (*M*. *septentrionalis*), and tricolored (*Perimyotis subflavus*) bats, and is caused by cutaneous infections with *Pseudogymnoascus destructans* (*Pd*). Big brown bats (*Eptesicus fuscus*) are highly resistant to *Pd* infections. Seven different fatty acids (myristic, pentadecanoic, palmitic, palmitoleic, oleic, and, linoleic acids) occur in the wing epidermis of both *M*. *lucifugus* and *E*. *fuscus*, 4 of which (myristic, palmitoleic, oleic, and, linoleic acids) inhibit *Pd* growth. The amounts of myristic and linoleic acids in the epidermis of *M*. *lucifugus* decrease during hibernation, thus we predicted that the epidermal fatty acid profile of *M*. *lucifugus* during hibernation has a reduced ability to inhibit *Pd* growth. Laboratory *Pd* growth experiments were conducted to test this hypothesis. The results demonstrated that the fatty acid profile of *M*. *lucifugus* wing epidermis during hibernation has a reduced ability to inhibit the growth of *Pd*. Additional *Pd* growth experiments revealed that: a) triacylglycerols composed of known anti-*Pd* fatty acids do not significantly affect growth, b) pentadecanoic acid inhibits *Pd* growth, and c) 1-oleoglycerol, which is found in the wing epidermis of *E*. *fuscus*, also inhibits the growth of this fungus. Analyses of white adipose from *M*. *lucifugus* also revealed the selective retention of oleic and linoleic acids in this tissue during hibernation.

## Introduction

White-nose Syndrome (WNS) is an emergent disease that has killed over 6,000,000 bats in the USA and Canada. Mass mortality with WNS was first found at 6 caves in central NY State during the winters of 2007-08/08-09. WNS has since spread to bat hibernation sites located in 31 U.S. states and 5 Canadian provinces. WNS produces over-winter mortality rates of 75–98% for these in the northeast: little brown (*Myotis lucifugus*), Indiana (*M*. *sodalis*) northern long-eared (*M*. *septentrionalis*) and tricolored (*Perimyotis subflavus*) bats [1]. A white fungus associated with WNS has been identified as *Pseudogymnoascus destructans* (*Pd*), and it grows on the muzzle, wings, and ears of bats during hibernation [2]. Histological analyses of affected *M*. *lucifugus*, *M*. *septentrionalis*, and *P*. *subflavus* revealed that *Pd* hyphae penetrate both the epidermis and dermis, consuming hair follicles, sebaceous and sweat glands [3]. The optimal temperature for the growth of *Pd* is 12.5–15.8°C [4], and it probably was introduced to North America [5]. Laboratory studies reveal that cutaneous infection with *Pd* is the cause of death in WNS-affected bats [6, 7].

Bats do not remain torpid throughout the hibernation season. Bouts of torpor last for days, interrupted by brief (< 2 h) periods of high metabolic rate and body temperature (T_b_), known as arousal episodes that account for 80–90% of all energy (fat) utilized during hibernation [8]. Bats infected with *Pd* arouse more frequently from torpor during hibernation, which leads to a premature depletion of body fat reserves prior to the availability of food, and subsequent death [9, 10]. Big brown bats (*Eptesicus fuscus*) hibernating in the same New York mines where *M*. *lucifugus* develop severe *Pd* infections: 1) have torpor bouts of normal duration, 2) do not develop extensive cutaneous *Pd* infections, and, 3) usually survive hibernation with some body fat reserves remaining [11].

The mammalian epidermis is the first line of defense against cutaneous fungal infections because fungal hyphae must initially invade it [12]. The epidermis is composed chiefly of specialized epithelial cells named keratinocytes that occur in 4 distinct strata; they are produced in the deepest stratum (the stratum basale), and migrate to the top stratum (the stratum corneum) as they age [13]. The lipids of the stratum corneum are a mixture of compounds from both the extracellular matrix secreted by the keratinocytes, and sebum produced by the sebaceous glands. The extracellular matrix contains free sphingosine bases, ceramides, cholesterol, and free fatty acids (FFAs), whereas the sebum is composed of triacylglycerols, diacylglycerols, FFAs, wax esters, squalene, cholesterol, and cholesterol esters [13, 14]. The epidermis of bats also contains cerebrosides and monoacylglycerols, which makes them unique among mammals [15, 16]. Some free fatty acids are known to have potent anti-microbial effects [17].

The wing epidermis of hibernating *E*. *fuscus* has almost twice the mean myristic (14:0), palmitoleic (16:1), and, oleic (18:1) acid contents as that of *M*. *lucifugus* [18]. Experiments with *Pd* cultures also revealed that myristic, palmitoleic, oleic, and linoleic (18:2) acids inhibit *Pd* growth [18]. Pentadecanoic acid (15:0) is also found in the epidermis of both *M*. *lucifugus* and *E*. *fuscus*_,_ accounting for up to 6.7% of all fatty acids [18]. We thus predict that *Pd* growth would also be inhibited by pentadecanoic acid. Mammals cannot synthesize fatty acids containing an odd number of carbon atoms, whereas some species of plants and bacteria are able [19].

After ~4 months of hibernation, the total lipid content of the wing epidermis from *M*. *lucifugus* has decreased by almost one-third, with corresponding reductions in the levels of myristic and linoleic acids [18]. We therefore predict that these changes in epidermal lipid composition reduce the ability of it to inhibit the growth of *Pd* on *M*. *lucifugus* during hibernation.

Linoleic acid is a polyunsaturated fatty acid (PUFA), and mammals can synthesize saturated and monounsaturated fatty acids, but not PUFAs. Most plant species produce linoleic acid, as well as some insects. When mammals consume PUFAs, they are incorporated into their cellular and storage lipids [19].

During late summer/early fall, the body fat content of little brown bats (*M*. *lucifugus*) increases from 7 to 27% body mass [20]. Triacylglycerols stored in white adipose tissue (WAT) are the primary energy source utilized during mammalian hibernation [21]. A triacylglycerol consists of a single glycerol molecule linked to 3 fatty acid molecules by ester bonds [19]. The only source of linoleic acid for epidermal lipid maintenance during hibernation is thus the WAT. The genes involved in the synthesis of saturated and monounsaturated fatty acids are down regulated during mammalian hibernation [22], thus the primary source of these fatty acids for epidermal maintenance during hibernation are those derived from WAT triacylglycerols as well. This reliance on fatty acids mobilized from WAT may be one of the factors that causes the epidermal lipid composition of *M*. *lucifugus* to change during hibernation.

Studies on sciurid rodents revealed that saturated fatty acids were selectively mobilized from WAT during hibernation, whereas oleic and linoleic acids were selectively retained in their WAT [23–25]. We thus predict that the WAT of hibernating *M*. *lucifugus* selectively mobilizes fatty acids in a similar manner, and this in turn leads to a corresponding change in epidermal fatty acid composition. We predict that the fatty acid profile of WAT from *M*. *lucifugus*: a) decreases in the proportion of saturated fatty acids, b) increases in the proportion of oleic acid, and, c) increases in the proportion of linoleic acid during hibernation.

Laboratory culture experiments with *Pd* maintained on media varying in lipid composition were conducted in order to both test our hypothesis on the effects fatty acid compositions, and to determine the influences of triacylglycerols as well as monoacylglycerols on *Pd* growth as well. The fatty acid compositions of WAT collected from free-ranging *M*. *lucifugus* and *E*. *fuscus* were also determined to test our hypotheses on the mobilization of fatty acids.

## Materials and methods

### Laboratory growth experiments with *P*. *destructans*

Isolates of *Pd* used in this study were previously cultured from affected bats in NY during February 2008 (American Type Culture Collection, ATCC MYA-4855). For each experimental media/temperature treatment examined, starter culture material was transferred using a sterile disposable inoculating needle to 3 evenly spaced locations on the surface of each of 4–12 experimental Sabouraud dextrose agar (SDA) plates, yielding 12–36 replicates for every experimental media/temperature combination. The skin temperature (T_skin_) of torpid *M*. *lucifugus* is normally 5–7°C during hibernation [8, 10], whereas the T_skin_ of torpid *E*. *fuscus* under similar conditions is 7.5–13.3°C [11]. Experiments were thus conducted at low (4.9–6.4°C) and high (10.1–10.4°C) ambient temperatures to simulate conditions on the skin of torpid *M*. *lucifugus* and *E*. *fuscus*. Each group of plates was incubated for 40–56 d. Growth was quantified by measuring the total surface area visible for each colony. The surface area of each colony (mycelium) was measured at 7 d intervals by capturing digital images of each culture plate with an UVP Chromato-Vue (Upland, CA, USA) model C-75 viewing cabinet. We then calculated the surface area of each photographed colony using ImageJ Version 1.34S software (NIH, Bethesda, MD, USA). Measurements of colony areas started once they were visible to the unaided eye, which was after 12–14 d of incubation at 10.1–10.4°C, and after 20–21 d of incubation at 4.9–6.4°C. Inoculated plates were sealed inside plastic containers with sterile paper towels moistened with sterile water to maintain a relative humidity of ~100% during incubation. A single iButton model DS1922L logger (Maxim Semiconductor, Dallas, TX, USA) was placed inside each container to measure ambient temperature (T_a_) at 1 h intervals throughout incubation.

Eight different growth experiments were performed, involving 1–2 different types of modified SDA media each, their compositions are listed in Table 1. The control treatment in all experiments consisted of plates that contained SDA media only. Experiments A, B, and C, contained enough FFAs added to SDA media to bring their total content to 0.25–2.0%, which was within the range of individual fatty acid contents previously reported for the wing epidermis of *M*. *lucifugus* and *E*. *fuscus* [18]. Experiment A was conducted to determine the effect of pentadecanoic acid (15:0) on the growth of *Pd*. Experiments B and C were conducted to test the hypothesis that the ability of the fatty acid mixture found in the wing epidermis of *M*. *lucifugus* to inhibit the growth of *Pd* decreases during hibernation. The same 7 FFAs were added to the SDA media for the fall and hibernation treatments. The relative proportion (% of all fatty acids) of each fatty acid type found in the wing epidermis of *M*. *lucifugus* just prior to the onset of hibernation (fall) was 8.0% myristic acid, 2.9% pentadecanoic acid, 30.0% palmitic acid, 19.2% palmitoleic acid, 5.3% stearic acid, 14.9% oleic acid and 6.6% linoleic acid, whereas these proportion were 4.8%, 6.7%, 35.0%, 27.7%, 1.9%, 19.0%, and 3.5%, respectively, after ~4 months of hibernation [18]. The media treatments in Experiment B contained these fatty acids in the relative proportions of observed during either fall or hibernation while keeping the total content to 200 mg/g, which is the fatty acid content of *M*. *lucifugus* wing epidermis during the fall. Experiment B thus only examined the effects of the changes in the relative proportions of fatty acids which occur during hibernation. The total amount of fatty acids found in the wing epidermis of *M*. *lucifugus* during hibernation is just 68% of fall levels [18]. Experiment C was conducted to determine the combined effects of both the changes in relative fatty acid proportions, and the decreases in overall fatty acid content (mg/g) that occurs during hibernation in *M*. *lucifugus* wing skin on the growth of *Pd*. The fall media in Experiment C thus had the same fatty acid content as in Experiment B (200 mg/g), whereas the hibernation media had a total fatty acid content of 136 mg/g, with a FFA mixture of 4.8% myristic acid, 6.7% pentadecanoic acid, 35.0% palmitic, 27.7% palmitoleic acid, 1.9% stearic acid, 19.0% oleic acid, and 3.5% linoleic acid. All fatty acids added to SDA media were in the free (unbound) form, > 99% pure, and obtained from the Sigma-Aldrich Chemical Co. (St. Louis, MO, USA).

**Table 1:** Media, ambient temperature (T_a_), and sample size (N) for the *P*. *destructans* growth experiments.

Experiment	Media Treatments	Low T_a_ (°C)	N	High T_a_ (°C)	N
A	Control	4.9	27	10.4	33
	0.25% Pentadecanoic Acid (15:0*)	4.9	39	10.4	27
	1.0% Pentadecanoic Acid (15:0)	4.9	27	10.4	33
B	Control	6.4	33	10.3	24
	Fall (Proportional)	6.4	33	10.3	33
	Hibernation (Proportional)	6.4	33	10.3	33
C	Control	6.2	33	10.3	36
	Fall (Absolute)	6.2	36	10.3	30
	Hibernation (Absolute)	6.2	24	10.3	24
D	Control	6.4	30	10.1	24
	0.25% Glyceryl Trimyristate	6.4	36	10.1	24
	0.5% Glyceryl Trimyristate	6.4	30	10.1	18
E	Control	6.4	30	10.4	36
	0.25% Glyceryl Tripalmitoleate	6.4	19	10.4	23
F	Control	6.1	30	10.3	12
	0.25% Glyceryl Trioleate	6.1	36	10.3	30
	0.5% Glyceryl Trioleate	6.1	30	10.3	36
G	Control	6.4	33	10.2	29
	0.25% Glyceryl Trilinoleate	6.4	33	10.2	24
H	Control	6.3	15	NONE	
	0.5% 1-oleoglycerol	6.3	24	NONE	
	0.5% Oleic acid (18:1)	6.3	24	NONE	

* The number left of the colon indicates the number of carbon (C) atoms, the number to the right denotes the number of C-C double bonds [19].

Triacylglycerols account for about 15% of epidermal lipids found in the wings of bats, and monoacylglycerols, which are esters of glycerol and a single fatty acid molecule, account for about 4% [16]. The effects of these lipids on the growth of *Pd* are not known. Experiments D—G thus examined the effects of triacylglycerols on *Pd* growth. The control media in each these experiments consisted of SDA only, with no added lipids (Table 1). All experimental treatments contained enough triacylglycerol (TAG) to bring the content to 0.25% and in some cases, 0.5% of the media. This is within the range of individual TAG concentrations found in bat wing epidermis [16]. All TAGs added to the media were >98% pure, and were obtained from Sigma Chemical Company (St. Louis, MO, USA). Only TAGs that were both: a) esters of fatty acids known to inhibit the growth of *Pd* in the FFA form, and, b) found in the wing epidermis of bats [18] were used in these experiments. A TAG consisting of a glycerol molecule bound to 3 myristic acid molecules (glyceryl trimyristate) was added in Experiment D, whereas Experiment E involved a TAG that was an ester of 3 palmitoleic acid (glyceryl tripalmitoleate) molecules (Table 1). Glyceryl trioleate, a TAG which contains 3 oleic acid molecules, was added to the media in Experiment F (Table 1), whereas glyceryl linoleate, an ester of 3 linoleic acid molecules and glycerol, was used in Experiment G (Table 1). Experiment H was conducted to determine the effects of a monoacylglycerol containing oleic acid (1-oleoglycerol) on the growth of *Pd*, because it is found in the wing epidermal lipids of *E*. *fuscus* [16]. One of the media treatments contained 0.5% free oleic (18:1) acid, whereas the other was 0.5% 1-oleoglycerol (Table 1), both of which were >98% pure and obtained from Sigma Chemical Company (St. Louis, MO, USA). This experiment was conducted at 6.3°C because oleic acid does not greatly inhibit *Pd* growth at T_a_ < 10°C [18].

### White Adipose Tissue (WAT) analyses

The current study involved WAT samples collected from the carcasses of *M*. *lucifugus* and *E*. *fuscus* that were captured and sacrificed for 2 previous studies [9, 11], thus no additional bats were used for this study. Studies on *M*. *lucifugus* and *E*. *fuscus* [9, 11] were conducted in strict accordance with recommendations listed in the Guide for the Care and Use of Laboratory Animals of the National (US) Institutes of Health. The protocols were approved by the Fordham University Institutional Animal Care and Use Committee (protocol numbers CF11-03, 12–01, and 12–02). Protocols were also approved by the New York State Department of Health Institutional Animal Care and Use Committee. The capture of live bats in NY was also conducted under a Scientific License to Collect (#1373) issued by the New York State Department of Environmental Conservation.

The WAT from 25 free-ranging *M*. *lucifugus* were analyzed for fatty acid composition. All bats were collected from the same 2 adjacent abandoned mines located in Ulster County, New York (N41°50.64’, W74°04.92’). The fall feeding/fattening period of both *M*. *lucifugus* and *E*. *fuscus* typically concludes by the end of October/early November at these sites, at which point hibernation begins. Both bat species typically end hibernation and begin emerging from these mines during the following April. Consequently, 11 *M*. *lucifugus* were collected while torpid on 23 October and 5 November, in order to determine WAT lipid composition during fall, near the onset of hibernation. A total of 14 additional *M*. *lucifugus* were collected from these same sites on 31 January and 26 February, during hibernation. A total of 7 hibernating *E*. *fuscus* were also collected from these sites during 31 January and 26 February, immediately sacrificed using an Isoflurane overdose upon capture, and stored at -20°C [9, 11]. These bats were collected in order to determine WAT lipid composition during hibernation.

A ~150 mg biopsy of WAT was later taken from each bat carcass from the caudal area. All lipids were then extracted from each WAT sample using a chloroform/methanol procedure [26]. The extracted lipids were trans-esterified using 1.0 methanolic HCl, producing fatty acid methyl esters [27]. Fatty acid methyl esters (FAMEs) were then identified and quantified using a Model 5890 gas-liquid chromatograph (Hewlett Packard, Palo Alto, CA, USA) fitted with a 30 m Model DB-23 capillary column (J&W Scientific Inc., Folsom, CA, USA) and a flame ionization detector. The column was initially held at 110°C for 3 min., then raised to 160°C at 20°C/min., and finally brought to 210°C at a rate of 4°C/min. The carrier gas was helium flowing at a rate of 30 cm^3^/min. Mixtures of known FAME standards (NHI-C, GLC -10, GLC, 40, GLC-50, GLC-70, and GLC-80) obtained from Supelco, Inc., (Bellefonte, PA, USA) were also analyzed to accurately obtain the retention time of each FAME type [28]. WAT samples from these bats were previously analyzed for linoleic and α-linolenic (18:3) acid levels [9]. The stomachs of 3 *M*. *lucifugus* collected during October still contained insects, and they were also analyzed for fatty acid composition since dietary lipids are neither digested nor absorbed in the stomach [29].

Mean colony areas at the end of each *Pd* growth experiment that contained 3 media types were compared between treatments within the same T_a_ group using a one-way ANOVA (General Linear Models) procedure in conjunction with Tukey’s Highly Significant Difference (HSD) Test. Mean WAT fatty acid compositions (% of all fatty acids) were compared between bat species/groups using the same methods. Experiments E and G involved only 2 media types each, due to limitations in the amounts of TAGs available. The results of these experiments were thus analyzed using a Student’s *t*-test. The data on stomach contents were not statistically compared to any other data due to the small sample size (N = 3) for this group. All statistical methods were performed using SYSTAT version 12.0 software. Significance level was set at p < 0.05 in each case.

## Results

### Laboratory growth experiments with *P*. *destructans*

**Fig 1:**
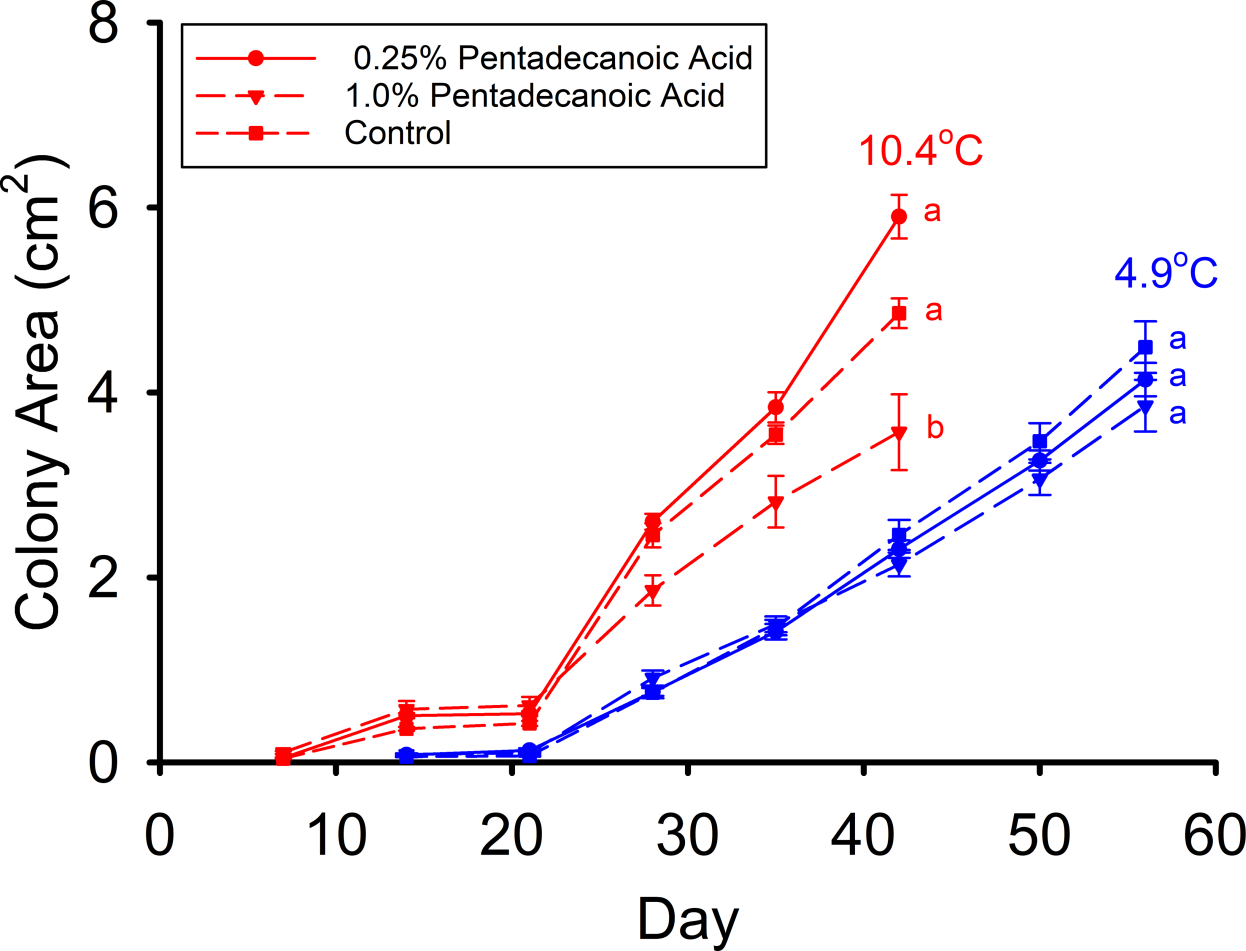
Mean (± SE) surface areas of *Pseudogymnoascus destructans* colonies at various stages of growth on control (squares), 0.25% pentadecanoic (15:0) acid (circles), and 0.5% pentadecanoic acid (triangles) SDA media at T_a_ = 4.9 (blue symbols) and 10.4°C (red symbols) in Experiment A. Means within the same T_a_ treatment sharing a common lower-case letter are not significantly different at the p < 0.05 level.

**Fig 2:**
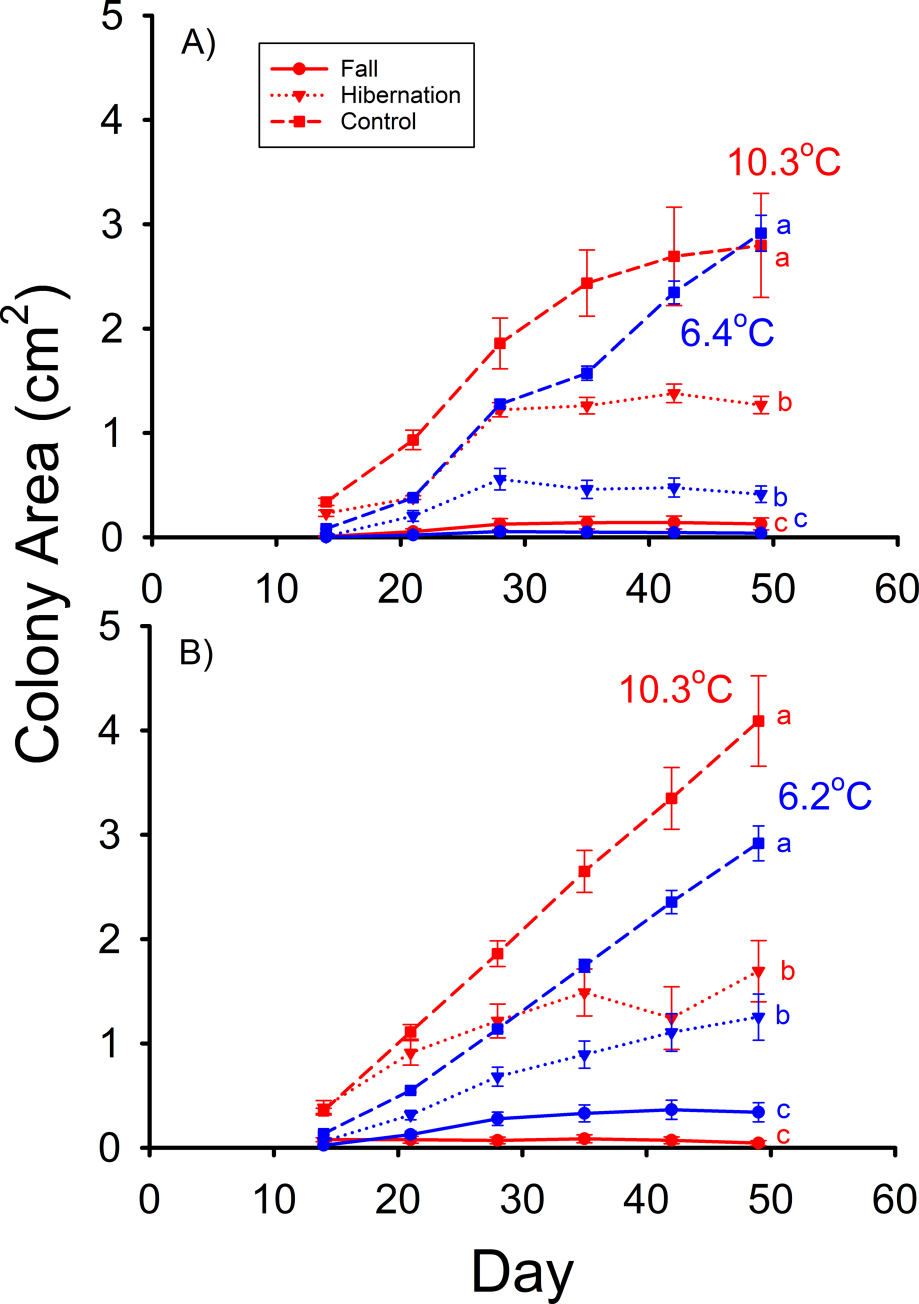
A) Mean (± SE) surface areas of *Pseudogymnoascus destructans* colonies at various stages of growth on the *M*. *lucifugus* fall FFA proportion (circles), hibernation FFA proportion (triangles), and control (squares) SDA media at T_a_ = 6.4 (blue symbols) and 10.3°C (red symbols). B) Mean (± SE) surface areas of *Pseudogymnoascus destructans* colonies at various stages of growth on the *M*. *lucifugus* fall FFA content (circles), hibernation FFA content (triangles), and control (squares) SDA media at T_a_ = 6.2 (blue symbols) and 10.3°C (red symbols). Means within the same T_a_ treatment sharing a common lower-case letter are not significantly different at the p < 0.05 level.

The mean colony (mycelium) area of the 0.25% pentadecanoic acid and control treatments were not significantly different from each other (Fig 1), and both were significantly greater than the area of the 1.0% pentadecanoic acid treatment at 42 d (F_2,36_ = 14.352, p < 0.001) in the high T_a_ group of Experiment A. At low T_a_ in Experiment A, no significant differences between the treatments were found (F_2,80_ = 0.327, p = 0.722) after 56 d (Fig 1). The mean *Pd* colony areas of both the fall and hibernation treatments were significantly less than that of the control after 49 d at 10.3°C1 (F_2,87_ = 29.110, p < 0.001) in Experiment B (Fig 2A). The mean colony area on the hibernation media was significantly greater than that of the fall treatment (Fig 2A). This same relative relationship for mean colony areas was observed between these treatments after 49 d of incubation at 6.4°C (F_2,96_ = 80.629, p < 0.001) in this experiment (Fig 2A). After 49 d of incubation at 10.3°C in Experiment C, the mean *Pd* colony areas of the fall and hibernation treatments were substantially less than that of the control (F_2,87_ = 41.769, p < 0.001). The mean colony area of the hibernation media was also significantly greater than that of the fall media (Fig 2B). Likewise, the mean *Pd* colony areas of the fall and hibernation treatments were less than the control after 49 d of incubation at 6.2°C (F_2,90_ = 78.427, p < 0.001). The mean colony area of the hibernation media was significantly greater than that of the fall media at 6.2°C (Fig 2B).

**Fig 3:**
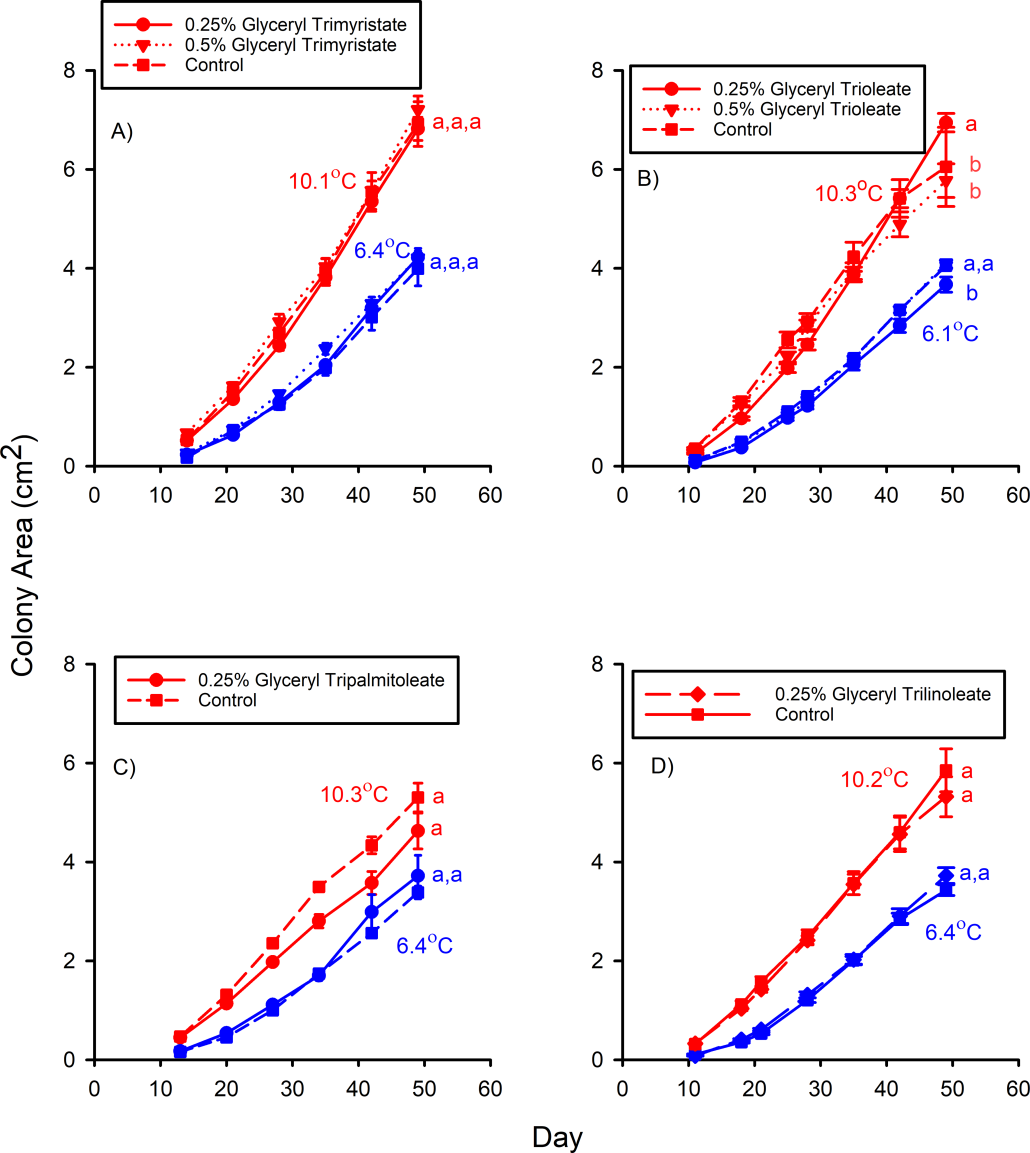
A) Mean (± SE) surface areas of *Pseudogymnoascus destructans* colonies at various stages of growth on 0.25% (circles), 0.5% glyceryl trimyristate (triangles), and control (squares) SDA media at T_a_ = 6.4 (blue symbols) and 10.1°C (red symbols). B) Mean (± SE) surface areas of *Pseudogymnoascus destructans* colonies at various stages of growth on 0.25% (circles), 0.5% glyceryl trioleate (triangles), and control (squares) SDA media at T_a_ = 6.1 (blue symbols) and 10.3°C (red symbols). C) Mean (± SE) surface areas of *Pseudogymnoascus destructans* colonies at various stages of growth on 0.25% glyceryl tripalmitoleate (circles) and control (squares) SDA media at T_a_ = 6.4 (blue symbols) and 10.3°C (red symbols). Means sharing a common lower-case letter are not significantly different at the p < 0.05 level. D) Mean (± SE) surface areas of *Pseudogymnoascus destructans* colonies at various stages of growth on 0.25% glyceryl trilinoleate (diamonds) and control (squares) SDA media at T_a_ = 6.4 (blue symbols) and 10.2°C (red symbols). Means within the same T_a_ treatment sharing a common lower-case letter are not significantly different at the p < 0.05 level.

**Fig 4:**
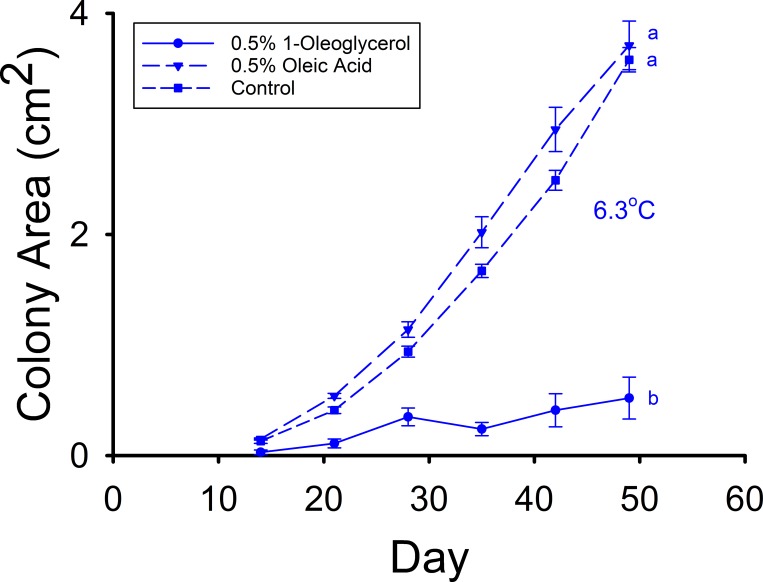
Mean (± SE) surface areas of *Pseudogymnoascus destructans* colonies at various stages of growth on 0.5% oleic (18:1) acid (triangles), 0.5% 1-oleoglycerol (circles), and control (squares) SDA media. Means sharing a common lower-case letter are not significantly different at the p < 0.05 level.

The mean *Pd* colony areas of the 0.25% and 0.5% glyceryl trimyristate treatments did not significantly differ from that of the control media after 49 d of growth at either 10.1°C (F_2,71_ = 0.324, p *=* 0.724) or 6.4°C (F_2,96_ = 0.280, *p* = 0.756) in Experiment D (Fig 3A). The mean colony areas of control and 0.5% glyceryl trioleate treatments were statistically equivalent after 49 d of growth at 10.3°C in Experiment F (Fig 3B), but both were slightly less than that of the 0.25% glyceryl trioleate treatment (F_2,75_ = 3.326, p = 0.041). The mean colony area of the 0.25% glyceryl trioleate media was slightly less than those of the control and 0.5% glyceryl trioleate media (F_2,93_ = 4.522, p = 0.013) after 49 d of growth at 6.1°C (Fig 3B), however. Likewise, the mean *Pd* colony of the 0.25% glyceryl tripalmitoleate treatment did not significantly differ from that of the control after 49 d at either 10.3°C (t = -1.454, df = 57, p *=* 0.076) or 6.4°C (t = 0.877, df = 50, p = 0.808) during Experiment E (Fig 3C). The addition of 0.25% glyceryl trilinoleate did not significantly affect *Pd* colony area relative to the control after 49 d of growth at either 10.2°C (t = -0.882, df = 52, p = 0.382) or 6.4°C (t = 1.525, df = 64, p = 0.132) in Experiment G (Fig 3D). In Experiment H, the control and oleic acid (18:1) treatments did not significantly differ in mean *Pd* colony area after 49 days of growth at 6.3°C (Fig 4), but the mean colony area of the 1-oleogylcerol treatment was substantially less than those for both of these groups (F_2,60_ = 90.825, p < 0.001).

### White Adipose Tissue (WAT) analyses

The same 9 fatty acids were found in all WAT samples. The 3 WAT groups did not significantly differ in the relative concentrations of lauric (12:0) acid (F_2,29_ = 1.771, p = 0.19), pentadecanoic acid (F_2,29_ = 0.347, p = 0.71), palmitic acid (F_2,29_ = 1.001, p = 0.38) or α-linolenic (18:3) acid (F_2,29_ = 2.398, p = 0.11; Table 2). The myristic acid level of WAT from *M*. *lucifugus* collected during the fall was more than twice that (Table 2) collected during hibernation (F_2,29_ = 13.288, p < 0.001), and was significantly greater than the myristic content of WAT collected from hibernating *E*. *fuscus* as well. The palmitoleic acid content of WAT from *M*. *lucifugus* during the fall was 2-3X greater than those of the WAT from *M*. *lucifugus* and *E*. *fuscus* (Table 2) during hibernation (F_2,29_ = 17.072, p < 0.001), and the level of palmitoleic acid in the WAT of *M*. *lucifugus* during hibernation was also greater than that of *E*. *fuscus* WAT (Table 2).

**Table 2:** Mean (± SE) fatty acid compositions of lipids from the white adipose tissue (WAT) and stomach contents of bats.

Fatty Acid Type*	Symbol**	Fall *M*. *lucifugus* WAT***	Hibernating *M*. *lucifugus* WAT	Hibernating *E*. *fuscus* WAT	*M*. *lucifugus* stomach contents
Lauric Acid	12:0	5.8 ± 2.3^a^	2.8 ± 1.72^a^	0.3 ± 0.2^a^	0.8 ± 0.6
Myristic Acid	14:0	7.2 ± 1.0^a^	3.3 ± 0.7^b^	0.6 ± 0.3^b^	19.0 ± 6.6
Pentadecanoic Acid	15:0	0.3 ± 0.2^a^	0.3 ± 0.2^a^	0.04 ± 0.04^a^	0.2 ± 0.2
Palmitic Acid	16:0	20.4 ± 0.6^a^	18.4 ± 1.1^a^	17.6 ± 2.8^a^	26.0 ± 6.6
Palmitoleic Acid	16:1	24.7 ± 2.5^a^	15.0 ± 1.3^b^	7.9 + 1.4^c^	18.8 ± 5.5
Stearic Acid	18:0	1.0 ± 0.3^a^	2.5 ± 0.4^b^	1.8 ± 0.4^b^	2.0 ± 0.9
Oleic Acid	18:1	22.8 ± 1.6^a^	36.4 ± 2.9^b^	49.4 ± 3.2^c^	22.4 ± 5.7
Linoleic Acid	18:2	10.1 ± 0.8^a^	13.7 ± 0.9^b^	18.3 ± 1.9^c^	7.5 ± 2.0
α-linolenic Acid	18:3	4.1 ± 0.4^a^	4.2 ± 0.6^a^	2.3 ± 0.8^a^	1.7 ± 0.8

*% of all fatty acids present in sample.

**The digit left of the colon is the number of C atoms; right is the number of C-C double bonds [19].

***Means sharing a common lower-case letter within the same fatty acid category are not significantly different at the P < 0.05 level. N = 11 for each fall (23 October– 5 November) *M*. *lucifugus* mean, 14 for each hibernating (31 January– 26 February) *M*. *lucifugus* mean, 7 for each hibernating (31 January– 26 February) *E*. *fuscus* mean, and 3 for each *M*. *lucifugus* stomach contents mean.

The mean stearic acid levels of WAT collected from both *M*. *lucifugus* and *E*. *fuscus* during hibernation (Table 2) were both greater than that of the *M*. *lucifugus* WAT in the fall (F_2,29_ = 4.954, p = 0.01). The mean level of oleic acid in *E*. *fuscus* WAT (Table 2) was nearly twice that found in both *M*. *lucifugus* WAT groups (F_2,29_ = 20.024, p < 0.001), and the mean level in *M*. *lucifugus* WAT during hibernation was significantly greater than that of the fall *M*. *lucifugus* WAT (Table 2). The mean linoleic acid content of *E*. *fuscus* WAT was significantly greater than those of both *M*. *lucifugus* groups (F_2,29_ = 11.796, p < 0.001), and that from hibernating *M*. *lucifugus* was significantly greater than during fall (Table 2), while the mean concentrations of α-linolenic acid (18:3) in WAT did not significantly vary (F_2,29_ = 2.398, p = 0.11). These same 9 fatty acid types were also found in the fall stomach contents of 3 *M*. *lucifugus* (Table 2).

## Discussion

The results of Experiment A clearly support the hypothesis that the growth of *Pd* is inhibited by pentadecanoic acid. The reduction in *Pd* growth, however, occurred only at the high (10.4°C) incubation temperature. Pentadecanoic inhibited *Pd* growth by about 50% after 42 d at 10.4°C, which is substantially greater than the level of inhibition observed when either myristic or stearic acids were added to the media at this T_a_ [18]. Integrating these findings with those previously obtained reveals that the relative strength of *Pd* growth inhibition are: linoleic acid > palmitoleic acid > oleic acid > pentadecanoic acid > myristic acid ≈ stearic acid, at T_a_ = 10.4–13.4°C. At T_a_ = 4.0–5.0°C, however, myristic, stearic, and pentadecanoic acids do not substantially affect *Pd* growth, and the inhibitory effect of oleic acid is greatly reduced [18]. These temperature effects may thus be one factor that makes *M*. *lucifugus* more susceptible to *Pd* infections, since the T_skin_ of this bat species is normally 5–7°C during hibernation, whereas the T_skin_ of hibernating *E*. *fuscus* is normally 7–13°C.

The results of Experiments B & C support the hypothesis that the changes in wing epidermal lipid composition that occur during hibernation reduce the ability of this tissue to inhibit *Pd* growth. At 6.2°C, the changes in the relative proportions of fatty acids alone decreased the ability of the fatty acid mixture to inhibit *Pd* growth (Fig 2A). When the changes in absolute fatty acid content were simulated in Experiment C, *Pd* growth at 6.4°C was reduced by only about half, relative to control (Fig 2B). These findings provide important insights into the progression of WNS during hibernation. Hibernating *M*. *lucifugus* typically do not exhibit severe *Pd* infections and subsequent WNS until about January [7, 10]. The results of Experiment C indicate that within the normal T_skin_ range of torpid *M*. *lucifugus*, the epidermal fatty acid composition of this species is sufficient to prevent the growth of *Pd* on the wings at the onset of hibernation, but during hibernation, it changes to the point where it permits substantial *Pd* growth. It thus appears that another factor that favors cutaneous infections with *Pd* on the wings of *M*. *lucifugus* during hibernation is the associated change in epidermal fatty acid composition.

The results of Experiments D through G reveal that triacylglycerols of fatty acids known to inhibit *Pd* growth when in the FFA form do not substantially affect *Pd* growth. Epidermal triacylglycerols may nevertheless have an important role in the defense against fungal growth by serving as a source of FFAs [17]. The findings of Experiment H reveal the opposite scenario for a monoacylglycerol, however. The addition of 0.5% oleic acid to the SDA media did not significantly affect *Pd* growth at T_a_ = 6.3°C (Fig 4), but the addition of 0.5% of a oleic acid monoacylglycerol (1-oleoglycerol) reduced *Pd* growth to just 9.7% of control after 49 d of incubation. These results suggest that monoacylglycerols in general may be more potent inhibitors of *Pd* growth than their FFA counterparts. Other monoacylglycerol types have been shown to have anti-fungal properties [30], and 1-oleoglycerol is 1 of 4 monoacylglycerols found in the wing epidermis of *E*. *fuscus* [16]. The anti-*Pd* properties of other epidermal monoacylglycerols, and their concentrations in *Myotis spp*., both warrant further investigation.

The same 9 fatty acids were found in the WAT of both *M*. *lucifugus* and *E*. *fuscus*, and they also appeared in the fall stomach contents of 3 *M*. *lucifugus* (Table 2). This indicates that the effects of the fall diet on both epidermal and WAT fatty acid composition warrant further investigation. These fatty acids except lauric and α-linolenic acids occur in the epidermis of both *M*. *lucifugus* and *E*. *fuscus* [18, 31]. Bats in temperate regions are insectivorous [32–34], thus the pentadecanoic, linoleic and α-linolenic acids found in the WAT of both *E*. *fuscus* and *M*. *lucifugus* are derived from their insect diets. Pentadecanoic acid is commonly found in the lipids of aquatic invertebrates [35]. The differences in both WAT and epidermal fatty acid compositions between *M*. *lucifugus* and *E*. *fuscus* thus may be in part due to corresponding differences in diet composition.

The relative proportion of myristic acid in *M*. *lucifugus* WAT decreased during hibernation (Table 2), which is consistent with the hypothesis that the relative proportion of saturated fatty acids decrease during hibernation. The relative proportions of lauric, pentadecanoic, and palmitic acids in the WAT of *M*. *lucifugus* did not significantly change during hibernation, however, and the fraction of stearic acid actually increased (Table 2). The relative proportion of most saturated fatty acids in the WAT of *M*. *lucifugus* thus do not decrease during hibernation, as they do in the WAT of hibernating rodents.

The relative proportion of oleic acid in the WAT of *M*. *lucifugus* almost doubled during hibernation (Table 2), and that of linoleic acid also increased, as predicted. The WAT of hibernating *E*. *fuscus* contained significantly more oleic and linoleic acids than that collected from hibernating *M*. *lucifugus* (Table 2), which suggests that the fall diet *E*. *fuscus* contains relatively more of these fatty acids. Fatty acid synthesis decreases during hibernation [24], thus the primary source of fatty acids for the epidermis during this period is probably the WAT. The low rate of mobilization of linoleic acid from WAT may be one reason why the epidermal content of it decreases during hibernation. Lauric acid does not appear in the epidermis of either *M*. *lucifugus* or *E*. *fuscus* [18, 31]. The mobilization of fatty acids from triacylglycerols stored in WAT of mammals is a selective process. Some fatty acid types tend to be mobilized more readily than others, leaving the remaining triacylglycerols enriched with other fatty acid types during the course of fasting. Studies on fasting laboratory rats revealed that the proportions of myristic and palmitic acids stored in the WAT triacylglycerols decreased, whereas the relative proportions of palmitoleic, oleic, and linoleic acids increase [36].

Hibernating rodents display a similar pattern of selective fatty acid mobilization from stored triacylglycerols when fasting during torpor. The relative proportions of palmitic and stearic acids in the WAT of hibernating thirteen-lined ground squirrels (*Ictidomys tridecemlineatus*) decreased during this period, while oleic and linoleic acids were selectively retained [25]. Our study reveals that oleic and linoleic acids are selectively retained in WAT of *M*. *lucifugus* during hibernation as well. The mechanisms behind this selective mobilization/retention of fatty acids are unknown. Hormone sensitive lipase is the major enzyme involved in the mobilization of fatty acids from stored triacylglycerols, and experiments with recombinant rat and human forms of this enzyme revealed that the activity of it cannot fully account for the selective mobilization/retention of fatty acids observed in whole adipocytes [37]. Further investigation of both lipid synthesis and mobilization during hibernation will undoubtedly provide a new understanding of *Pd* susceptibility, as well as mammalian lipid biochemistry in general.
